# Evaluation of Neutrophil–Lymphocyte Ratio, Platelet–Lymphocyte Ratio and Red Blood Cell Distribution Width–Platelet Ratio for Diagnosis of Premature Ovarian Insufficiency 

**Published:** 2016-12

**Authors:** Gulsah Ilhan, Fatma Ferda Verit Atmaca, Esra Altan, Ali Galip Zebitay, Hamdullah Sozen, Hurkan Akyol, Meryem Kurek Eken

**Affiliations:** 1 Department of Obstetrics and Gynecology, Division of Reproductive Endocrinology and Infertility, Suleymaniye Research and Education Hospital, Istanbul, Turkey; 2 Department of Obstetrics and Gynecology, Division of Reproductive Endocrinology and Infertility, Adnan Menderes University, Aydın, Turkey

**Keywords:** Inflammation Marker, Premature Ovarian Insufficiency

## Abstract

**Objective:** To evaluate whether systemic inflammatory markers (neutrophil to lymphocyte ratio (NLR), platelet to lymphocyte ratio (PLR) and red blood cell distribution width (RDW) to platelet ratio (RPR)) can be used as reliable markers for the diagnosis of premature ovarian insufficiency (POI) and to determine if there is a relationship between these markers and follicle stimulating hormone (FSH), Anti-Müllerian Hormone (AMH) levels.

**Materials and methods:** Written and electronic medical records were reviewed using searches for diagnoses with the terms of 'premature ovarian failure', 'premature ovarian insufficiency'. Patients younger than the age of 40 were diagnosed to have premature ovarian insufficiency based on their menstrual history and sonographic examination and they were compared with healthy females. Complete blood counts, day-3 hormone profiles, AMH levels of all subjects were analyzed.

**Results:** NLR was statistically higher in POI group compared with controls (p < 0.05). NLR had a positive correlation between FSH (r = 0.23, p = 0.045) and a negative association with AMH (r = - 0.27, p = 0.018). The area under ROC curve for NLR in POI was 0.66, with a threshold value 1.5 and sensitivity = 75.7 % and specificity = 46.0 %.

**Conclusion:** NLR can be a marker for the diagnosis of POI. There is a close relationship between NLR and ovarian reserve markers such as FSH and AMH.

## Introduction

Premature ovarian insufficiency (POI) is an enigmatic condition which is still not completely understood. POI is also called primary ovarian failure and affects 1% of women under the age of 40 years (1, 2). The disease can result from ovarian dysgenesis, follicle growth arrest, premature depletion of the follicle pool or follicular atresia. In patients with POI, ovarian histological features are very similar to those found in post-menopausal ovaries resulting from the natural process of follicular atresia (3).

POI is a term to define the women younger than 40 years of age who present with at least a four-month history of amenorrhea and hypergonadotropic serum profile (follicle stimulating hormone (FSH) levels ≥ 40 mIU per milliliter) (4-8). A diverse etiology for POI has been postulated. Although infectious agents, pelvic surgery, chemotherapy, autoimmune disease, genetic problems, environmental factors may lead to POI, a majority of cases are idiopathic (9).

POI is characterized by sex steroid deficiency (1). It is well established that estrogens influence inflammatory and immune processes. Antiinflammatory activity of estrogen is executed by inhibiting many proinflammatory pathways of innate immunity, adaptive immunity, and inflammatory tissue responses. Decrease of estrogen level leads to a shift towards to a proinflammatory direction (10).

Neutrophil to lymphocyte ratio (NLR), platelet to lymphocyte ratio (PLR) and red blood cell distribution width (RDW) to platelet ratio (RPR) correlate with the markers of pro-inflammatory state that are widely known as the hematological markers of systemic inflammation. NLR was shown to be elevated in miscellaneous diseases and has been widely used to determine the severity of inflammation (11-18). PLR was found to be an independent risk factor of reduced survival in patients with various kinds of malignancies (19-22). RPR was concluded as a valuable, novel labarotory test to predict mortality in various diseases (23, 24).

Current studies have failed to determine specific biomarkers of POI. We also have limited tool for diagnosis. It is hypothesized that estrogen defiency in POI patients leads to a shift towards to an inflammatory direction and widely known markers of systemic inflammation including NLR, PLR and RPR might be affected.

The aims of this study were to evaluate whether inflammatory markers canbe used as reliable markers for diagnosis of POI and to determine if there is any relationship between these markers and ovarian reserve markers such as FSH, Anti-Müllerian Hormone (AMH) levels.

## Materials and methods

We conducted a retrospective study to analyze women presenting with POI. This study was carried out at Suleymaniye Maternity and Women’s Disease Education and Research Hospital, In Vitro Fertilization clinic, Istanbul, Turkey. Written and electronic medical records between January 2013 and February 2016 were reviewed using searches for diagnoses with the terms of 'premature ovarian failure', 'premature ovarian insufficiency'. All procedures performed in studies involving human participants were in accordance with the ethical standards of institutional and/or national research committee and with the 1964 Helsinki decleration and its later amendments or comparable ethical standards.

The study was approved by local ethics committee. (the ethical code number: 87534341/846).

37 patientswith POI (Group A) and 37 healthy females (Group B) were included. Patients younger than the age of 40 were diagnosed to have premature ovarian insufficiency based on their menstrual history (at least a 4-month history of amenorrhea), gonadotropins levels (follicle stimulating hormone (FSH) levels ≥ 40 mIU per milliliter) and sonographic examination (TOSHIBA EXPERT 5 MHz) (no or a few demonstrable follicles on transvaginal ultrasound) were included in this study. All POI patients had a normal female 46,XX karyotype.

Secondary causes of amenorrhea including pregnancy, polycystic ovarian syndrome, chronic medical illness(uncontrolled diabetes mellitus or celiac disease), hypothalamic amenorrhea, extreme exercise, poor caloric intake, hyperprolactinemia, hyperthyroidism, hypothalamic or pituitary lesions, women with histories of chemotherapy, pelvic surgery, radiation exposure or premature ovarian insufficiency due to extensive ovarian surgery were excluded.Medical conditions that may interfere with complete blood count parameters including hematologic, cardiovascular, kidney-liver disease, asthma, arthritis, neoplastic disease such as androgen-secreting tumours, ovarian tumour, use of glucocorticoids, infectious and parasitic diseases were also excluded. 

Complete blood counts, day-3 hormone profiles, AMH levels of all subjects were analyzed. NLR, PLR and RPR were calculated for both the patients and the control group.The complete blood count was performed using an auto haematology analyser (BC-6800, Mindray). Plasma levels of FSH were measured by conventional radioimmunoassay (Immunotech Beckman, France) and AMH was measured by ELISA (Enzyme-Linked ImmunoSorbent Assay) (*Beckman Coulter-Inc*). 

In statistical anaylsis, SPSS 17.0 for windows package program was used. Mean and standart deviation values of parameters were used to describe scale variables. Kolmogorov Smirnov test was used to define normality of parameters. Independent Samples T-test was usedfor normally distributed parameters. Mann Whitney U test was used for nonparametric values (Neutrophile, RDW and FSH). Pearson's correlation test was used for correlation between variables, and linear regression was used to analyze estimated model. ROC analysis was usedfor determination of predictive value of NLR. Difference analysis was performed at 95% Confident Interval and correlation analysis was performed at 95-99% Confident Interval.

p < 0.05 was considered statistically significant.

## Results

A total of 37 patients meeting the strict inclusion and exclusion criteria were included in Group A and 37 healthy subjects were included in Group B. Baseline characteristics of both groups were shown in Table 1. There were no differences in terms of age, neutrophile, lymphocyte, platelet count, and RDW (p > 0.05, for all). However, FSH levels were significantly higher and AMH levels were lower in POI group (p < 0.0001, for all) (Table 1).

Although there were no statistically significant differences on neutrophile and lymphocyte counts, NLR showed statistically significant difference between groups (p < 0.05) (Table 2).

**Figure 1:**
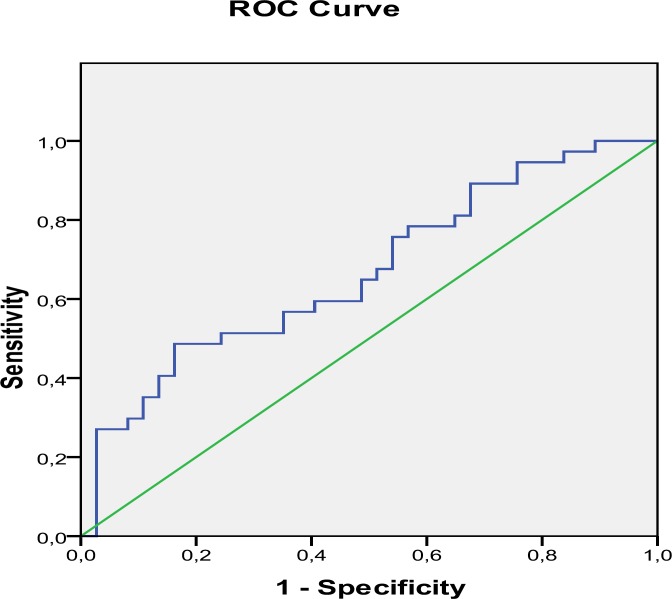
ROC Curve analysis for NLR

The correlation between NLR and ovarian reserve markers such as FSH and AMH were also analyzed. NLR had a positive correlation between FSH (r = 0.23, p = 0.045) and a negative association with AMH (r = -0.27, p = 0.018). However, linear regression analysis found that FSH and AMH levels were not independent variables that were associated with NLR (p > 0.05, for both). We also performed ROC curve analysis for NLR in POI group that was shown in Figure1. The area under ROC curve for NLR in POI was 0.66, with a threshold value 1.5 and sensitivity = 75.7 % and specificity = 46.0 %.

## Discussion

In the present study, we found that only NLR, but not PLR or RPR was statistically higher in POI group compared with controls. There was a close relationship between NLR and ovarian reserve markers such as FSH and AMH. Furthermore, ROC analysis showed that NLR may be considered a marker for the diagnosis of POI.

It is knowledged that estrogens influence inflammatory and immune processes. It has been shown that important proinflammatory pathways such as TNF, IL-1, IL-6, MCP-1, iNOS expression, production of MMPs, and activity of natural killer cells and reactive oxygen species formation are inhibited and antiinflammatory pathways such as IL-4, IL-10, TGF, TIMP, osteoprotegerin are stimulated by estrogen (10).

Some immunological alterations have been expressed in POI patients. For instance; organ specific autoantibodies can be detected up to 20% of POI cases (25). 

**Table 1:** Baseline characteristics of patient and control groups

**Parameters (Mean ± SD)**	**Group A (n = 37)**	**Group B (n = 37)**	**p**
Age (year)	33.38 ± 4.61	31.81 ± 3.76	0.113^a^
Neutrophile count (10e3/uL)	4.13 ± 1.17	3.75 ± 1.30	0.051^b^
Lymphocyte count (10e3/uL)	2.03 ± 0.67	2.24 ± 0.62	0.167^a^
Platelet count (10e3/uL)	254.70 ± 51.78	267.81 ± 63.15	0.332^a^
RDW (%)	41.61 ± 3.07	42.69 ± 4.20	0.239^b^
FSH (mIU/ml)	54.46 ± 16.89	7.19 ± 1.29	< 0.001^b^
AMH (ng/ml)	0.410 ± 0.43	1.45 ± 0.29	< 0.001^a^

AMH: Anti-müllerian Hormone, FSH: Follicle stimulating hormone, RDW: Red cell distribution width.

a Independent Samples T-test;

b Mann Whitney-U test

**Table 2:** Blood parameters of patient and control groups

**Parameters (Mean ± SD)**	**Group A (n = 37)**	**Group B (n = 37)**	**p**
NLR	2.20 ± 0.81	1.76 ± 0.64	0.011^a^
PLR	138.07 ± 51.35	125.71 ± 33.95	0.226^a^
RPR	0.17 ± 0.04	0.17 ± 0.05	0.880^a^

NLR: neutrophil to lymphocyte ratio, PLR: platelet to lymphocyte ratio, RPR: red blood cell distribution width to platelet ratio;

a Independent Samples T-test

Alteration in cellular immunity such as macrophage and dendrite cells abnormalities, change in CD4+/CD8+ ratio, as well as inappropriate expression of class II MHC antigens by granulosa cells have been seen (26).

NLR, PLR and RPR correlate with the markers of pro-inflammatory state that are widely known as the hematological markers of systemic inflammation. NLR has been widely used to determine the severity of inflammation (11-18). Up to date, it has been shown that diabetes mellitus, thyroid functional abnormalities, renal failure, essential hypertension, metabolic syndrome, valvular heart diseases, acute pancreatitis in pregnancy, cardiovascular disease, autoinflammatory diseases and malignancies including renal cell cancer, ovarian, lung, gastric and colorectal cancer may potentially affect the NLR (11-22). PLR was found to be an independent risk factor of reduced survival in patients with malignancies such as pancreatic and colorectal cancer (19-22). RPR is concluded as a valuable, novel labarotory test to predict mortality in acute pancreatitis, hepatic fibrosis and cirrhosis (23, 24). In our study; we assessed NLR, PLR and RPR as the predictors of disease in women with POI. We found that NLR showed statistically significant difference between groups, but the others not. ROC analysis showed that NLR can be a marker for the diagnosis of POI.

AMH is a member of TGF-B (transforming growth factor-B) family. It is an important ovarian aging test for the evaluation of follicular pool and has a strong correlation with antralfollicle count (27, 28). It is now widely considered the best laboratory test to represent total ovarian reserve (29-35). According to the results of the study conducted by Alipour et al (36), AMH serum level is more sensitive than FSH serum level in early diagnosis of POI. Also AMH has more negative predictive value. AMH is reported to be more useful in early diagnosis of POI. In our study; although there were statistically significant correlations between NLR and FSH and AMH, according to regression analysis, FSH and AMH levels are individually not predictors of NLR.

However, a new predictor of the disease is defined in the present study, this study has some limitations. It has a retrospective design and only represents experience of a single institution. Case group is small in number and the etiology is purely idiopathic.It is a hospital based study, so this study can not reflect all population. The results should be confirmed with larger groups.Retrospective design of the study did not allow to assess other systemic inflammatory markers including ILs, CRP and TNF-α.On the other hand, the systemic inflammatory response measured using the NLR, PLR and RPR has been proposed to be an inexpensive, widely available and reproducible way. Easier and these kind of cheaper tests will improve its use in clinical practice.

In conclusion, the main aim of our study is to identify a novel marker for POI and to have an idea aboutthe relation of this marker with ovarian reserve markers such as FSH and AMH in this group. We claimed that NLR may be used as a marker of POI. The low cost, easy accessibility and reproducibility of a NLR may promote its use in clinical practice.Further larger randomized controlled trials are needed for routine use in the future.
